# Predictive robot eyes enhance attentional guidance in cooperative human–robot interaction

**DOI:** 10.1038/s41598-025-19497-3

**Published:** 2025-09-23

**Authors:** Lara Naendrup-Poell, Linda Onnasch

**Affiliations:** Psychology of Action and Automation, Technische Universität Berlin, 10587 Berlin, Germany

**Keywords:** Human–robot interaction, HRI, Anthropomorphism, Eye-tracking, Predictive cues, Industrial HRI, Psychology, Human behaviour

## Abstract

A key factor in successful human–robot interaction (HRI) is the predictability of a robot’s actions. Visual cues, such as eyes or arrows, can serve as directional indicators to enhance predictability, potentially improving performance and increasing trust. This study investigated the effects of predictive cues on performance, trust, and visual attention allocation in an industrial HRI setting. Using a 3 (predictive cues: abstract anthropomorphic eyes, directional arrows, no cue) $\times$ 3 (experience in three experimental blocks) mixed design, 42 participants were tasked with predicting a robot’s movement target as quickly as possible. Contrary to our expectations, predictive cues did not significantly affect trust or prediction performance. However, eye-tracking revealed that participants exposed to anthropomorphic eyes identified the target earlier than those without cues. Interestingly, participant’s self-reports showed infrequent use of the cues as directional guidance. Still, greater cue usage, as indicated by fixation data, was associated with faster predictions, suggesting that predictive cues, particularly anthropomorphic ones, guide visual attention and may improve efficiency. These findings highlight the nuanced role of predictive cues in HRI: even when not heavily relied on or reflected in performance, they can subtly guide attention and support interaction.

## Introduction

In line with the vision of Industry 5.0, the industrial landscape is undergoing a transformation toward more adaptive and human-centric production environments^1^. A key driver of this shift is the integration of collaborative robots (CoBots) into human workspaces^2^. According to the World Robotics Report^3^, nearly every tenth industrial robot installed in 2022 was a CoBot - a robot designed to operate in physical and temporal proximity to humans, enabling joint tasks^4^. This development offers increased flexibility and autonomy^4^, but also introduces greater uncertainty^5,6^ and reduced predictability^7^. Predictability, however, is a key prerequisite for successful joint action: humans must be able to anticipate what the robot will do (i.e. the intention), when, and where an action is going to be performed^8^.

A promising approach to enhance predictability is the use of anthropomorphic design features, which are believed to increase perceived intentionality and activate familiar human-human interaction schemas that facilitate intuitive interaction^9–11^. According to Onnasch and Roesler^12^ anthropomorphism can be expressed through appearance, verbal or non-verbal communication, movement or context information. While many studies have highlighted the positive effects of anthropomorphic design on human–robot interaction (e.g.,^13–17^), recent research has also revealed potential drawbacks. Anthropomorphic design can undermine perceived reliability^18^, reduce trust in task-related settings^19^, decrease prosocial behavior^20^, or foster excessive emotional attachment^14^. The effectiveness of anthropomorphic design appears to depend on various moderating factors^9,21,22^. One of them is the application context^9,23^. Although positive effects are well documented in social settings, the impact in industrial settings remains ambiguous^9^. Furthermore, research on the so-called “matching hypothesis”^24^ also suggests that the appearance of a robot should match the sociability of a task, meaning that in contexts with traditionally lower sociability, like industrial applications, an anthropomorphic robot might not be seen as a good match. In general, regardless of the application context, anthropomorphic features should serve a clear, task-relevant purpose rather than being purely decorative^9^.

One task-relevant application of anthropomorphic cues is the use of gaze to communicate the robot’s intention and coordinate actions between robot and human^4^. In human interaction, gaze direction serves as a critical non-verbal communication tool^25^, allowing individuals to infer others’ focus of attention and predict their subsequent actions^26^. Humans are evolutionarily attuned to gaze^25^, and gaze cueing (the automatic tendency to follow others’ eye movements) is a well-documented phenomenon^27,28^. Apart from eyes, non-social stimuli like arrows might also trigger automatic gaze shifts in humans^29,30^, though some research suggests that arrows only allow for voluntary gaze shifts^31^. In an industrial context, where we might want to avoid overly anthropomorphic elements, arrows could be an option to guide people’s attention. Onnasch and colleagues^26,32,33^ investigated different predictive cues - photorealistic human eyes, abstract anthropomorphic eyes, and arrows. The arrows served as a non-anthropomorphic, directional cue, while the abstract anthropomorphic eyes were designed to tap into the positive effects of the human gaze mechanism while not being overly anthropomorphic to avoid negative effects in the industrial context. Their results confirmed that the abstract eyes led to a faster action prediction. Other studies have also confirmed that robot gaze cues improve action coordination and predictability^34,35^.

In uncertain environments where predictability and reliability are particularly important, another crucial factor for successful HRI is trust. Trust is commonly defined as the “attitude that an agent will help achieve an individual’s goal in a situation characterized by uncertainty and vulnerability” (^36^, p.51). Anthropomorphic design has been shown to have a positive effect on trust^9,37^, but trust is also shaped by the robot’s performance, including predictability and reliability of the robot’s behavior^38,39^.

Trust is therefore a dynamic attitude^39^ that evolves over time with three relevant phases: trust formation (where trust is built), trust dissolution (where trust decreases after violations, such as errors), and trust restoration (where trust may increase again, although possibly not to its original level)^40^. Introducing predictive cues holds promise for improving predictability and trust but also creates the potential for new errors. It is therefore important to examine not only their benefits but also their effects when they fail. Empirical research has confirmed that errors negatively affect perceived trust(worthiness)^41–43^. Particularly in systems with high anthropomorphism, which elicit stronger perceptions of intentionality^10^, errors can lead to decreases in perceived trust and reliability^5^. Nonetheless, anthropomorphism may also promote forgiveness, potentially mitigating some of the negative effects of errors^43,44^.

In summary, predictive cues - both anthropomorphic and non-anthropomorphic - hold potential for enhancing action predictability and trust in robots. Yet, it remains unclear how they affect performance and trust, particularly when errors occur.

Based on the existing literature, we hypothesize that predictive cues, especially anthropomorphic ones, improve performance compared to no cues as they provide additional directional information. However, when predictive cues lead to errors, we expect performance to decline relative to a condition without predictive cues. Nonetheless, we anticipate that once the interaction resumes without further errors, the benefits of predictive cues - particularly anthropomorphic ones - will reemerge, resulting in improved performance.

For trust, we expect predictive cues, especially anthropomorphic ones, to increase trust relative to no cues because they are thought to increase predictability. However, when predictive cues fail, we expect a trust dissolution leading to lower trust compared to having no predictive cues and therefore also no error. The effect of the failure is expected to be especially pronounced for the anthropomorphic cues. In line with the trust restoration phase, we predict an increase of trust after failure-free interaction. However, we expect it to remain lower for conditions with predictive cues than for those without. Since anthropomorphism has been suggested to facilitate forgiveness, we further anticipate a stronger post-failure trust recovery for anthropomorphic cues.

Although performance and trust are central outcome measures, understanding how predictive cues are actually used in practice is equally important. To gain deeper insight into participants’ cue usage, we analyzed their visual attention allocation using eye tracking. Eye movements offer valuable information about attentional processes^45,46^ and are particularly relevant for ensuring safe and efficient human–robot interaction^5^. Eye tracking has been widely used in HRI research for various purposes, including measuring engagement^47^, assessing trust^48–50^, evaluating gaze cue usage^35^, and analyzing visual attention allocation during interaction^5^. Given that predictive cues are designed to guide attention and facilitate action coordination, we explored whether participants actively relied on them by examining their fixation behavior during interaction. Since research on predictive cue usage in industrial settings remains limited, we implemented eye tracking as an exploratory measure.

In our experimental paradigm, participants were instructed to predict the movement target of the industrial CoBot Sawyer as quickly and accurately as possible. The CoBot’s arm moved toward one of six potential targets, which participants then had to select on a tablet placed in front of them. After selecting a target, participants completed a time-constrained memory task on the tablet. The earlier participants predicted the robot’s movement target, the more time they had for the memory task. Depending on the condition, Sawyer’s display showed either anthropomorphic cues (eyes), non-anthropomorphic cues (arrows), or no predictive cues. The predictive cues (both eyes and arrows) indicated the upcoming target by focusing on it both prior to and during the robotic arm’s movement. These visual cues were adapted from previous studies^26,32,33^ and were implemented as a between-subjects factor. While the cues functioned reliably in most trials, we deliberately introduced error trials in which the predictive cues misdirected attention by “looking” at a target opposite to the actual arm movement target. The trials were organized into three consecutive experimental blocks: the first block contained only correctly cued trials, the second block additionally included two error trials alongside correctly cued trials, and the third block again consisted solely of error-free trials. The experimental blocks were implemented as a within-subjects factor. Statistical analyses were conducted blockwise, differentiating between the first half and the second half per block. For further details on the design and variables, please refer to the Methods section. For a demonstration video of correctly cued trials and error trials see the OSF.

## Results

### Manipulation check and control variables

Perceived anthropomorphism was assessed as a manipulation check, while the individual tendency to anthropomorphize robots and mind perception were measured as control variables. Overall, perceived anthropomorphism was rated low (e.g., in terms of appearance: $M_{Eyes} = 12.4$, ($SD = 9.4$); $M_{Arrows} = 9.48$, ($SD = 12.5$); $M_{NoCue} = 8.90$, ($SD = 13.5$); on a scale from 0 to 100), without any significant differences between conditions in terms of appearance ($F(2,39) = 0.35$, $p = .70$, $\eta ^2_{p} = 0.02$), movement ($F(2,39) = 1.31$, $p = .28$, $\eta ^2_{p} = 0.06$) or context ($F(2,39) = 0.29$, $p = .75$, $\eta ^2_{p} = 0.01$). Participants did also not differ in terms of their tendency to anthropomorphize robots ($F(2,39) < 0.01$, $p > .99$, $\eta ^2_{p} < 0.01$) or in terms of mind perception ($F(2,39) = 0.42$, $p = .66$, $\eta ^2_{p} = 0.02$). Interestingly, more than two thirds of participants in the predictive cue conditions reported never or only sometimes using the cues to predict the robot’s target (11/14 in the eyes condition and 9/14 in the arrows condition), with only one person in the eyes condition stating they always used them.

Additionally, only 9 participants across both predictive cue conditions noticed the robot’s faulty predictive behavior during the second block (two arm movements with misleading cues), while 16 participants reported not noticing any errors, 6 were unsure, and 11 mentioned the robotic arm movement as faulty since it was not perceived as being straight to the target.

### Performance

#### Prediction speed and accuracy

**Figure 1:**
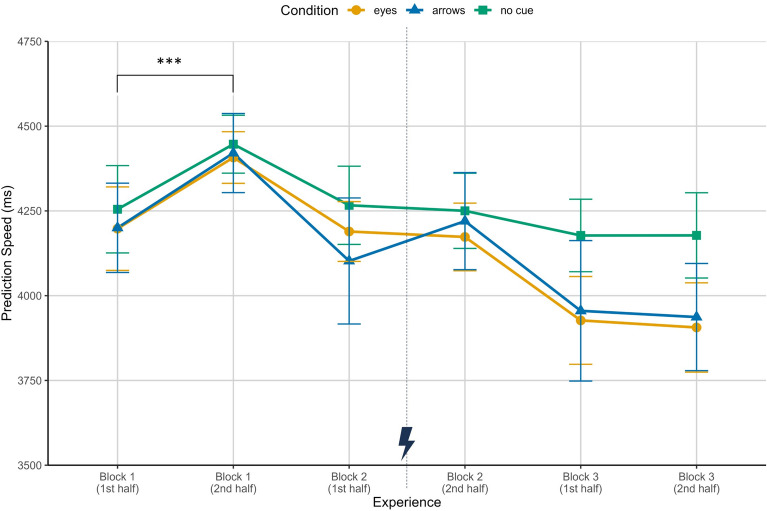
Prediction speed for selecting the correct targeted square in milliseconds over the three experimental blocks, each split in the first six trials and the last six trials, for the conditions eyes, arrows and no cue. Depicted are means and standard errors. The asterisks indicate a significant experience effect within block 1 ($p < . 001$). The lightning bolt icon marks the occurrence of cue error trials.

For prediction speed it can be seen that it initially increased within block 1 and then decreased (i.e. improved) almost consistently in all conditions (see Fig. 1). Descriptively, participants were slightly slower in the no cues condition. In block 3, for instance, participants in the eyes condition took on average about 3916 ms $$(SD = 489$$ms) to select the correct target, while participants in the arrows condition took 3946 ms ($$SD = 683$$ms) and participants without cues were the slowest with on average 4178 ms ($$SD = 435$$ms). However, no significant differences between cue conditions emerged in neither of the blocks (Block 1: $$F(2,39) = 0.07$$, $$p = .937$$, $$\eta ^2_{p} < 0.01$$; Block 2: $$F(2,39) = 0.17$$, $$p = .841$$, $$\eta ^2_{p} = 0.01$$; Block 3: $$F(2,39) = 0.99$$, $$p = .382$$, $$\eta ^2_{p} = 0.05$$). The analyses did only reveal a significant main effect of experience in the first block showing an increase in prediction time, i.e. slower responses ($$F(1,39) = 13.70$$, $$p < . 001$$, $$\eta ^2_{p} = 0.26$$). No other significant effects emerged within the blocks (Block 1: interaction effect, $$F(2,39) = 0.02$$, $$p = .978$$, $$\eta ^2_{p} < 0.01$$; Block 2: experience effect, $$F(1,39) = 0.49$$, $$p = .490$$, $$\eta ^2_{p} = 0.01$$, interaction effect, $$F(2,39) = 1.20$$, $$p = .313$$, $$\eta ^2_{p} = 0.06$$; Block 3: experience effect, $$F(1,39) = 0.15$$, $$p = .698$$, $$\eta ^2_{p} < 0.01$$, interaction effect, $$F(2,39) = 0.04$$, $$p = .961$$, $$\eta ^2_{p} < 0.01$$). In terms of accuracy, a significant increase was found within block 1 ($$F(1,39) = 22.51$$, $$p < .001$$, $$\eta ^2_{p} = 0.37$$), but no significant differences emerged across predictive cue conditions (Block 1: $$F(2,39) = 0.15$$, $$p = .858$$, $$\eta ^2_{p} < 0.01$$; Block 2: $$F(2,39) = 1.45$$, $$p = .246$$, $$\eta ^2_{p} = 0.07$$; Block 3: $$F(2,39) = 1.60$$, $$p = .216$$, $$\eta ^2_{p} = 0.08$$). In general, accuracy was rather high with the lowest value of $$M = 0.77$$ ($$SD = 0.16)$$ in the arrows condition in the beginning of block 1 and values of consistently above 0.90 in block 3 for all conditions. All other effects regarding accuracy were not significant (Block 1: interaction effect, $$F(2,39) = 1.76$$, $$p = .185$$, $$\eta ^2_{p} = 0.08$$; Block 2: experience effect, $$F(1,39) = 0.62$$, $$p = .438$$, $$\eta ^2_{p} = 0.02$$, interaction effect, $$F(2,39) = 1.19$$, $$p = .314$$, $$\eta ^2_{p} = 0.06$$; Block 3: experience effect, $$F(1,39) = 0.07$$, $$p = .788$$, $$\eta ^2_{p} < 0.01$$, interaction effect, $$F(2,39) = 0.51$$, $$p = .602$$, $$\eta ^2_{p} = 0.03$$).

#### Performance in memory task

Regarding the performance in the memory task, no significant differences between the predictive cue conditions, nor any significant experience or interaction effects were observed. For detailed test statistics see Table 1. In general, the performance was quite good with the lowest performance rate of $M_{Arrows} = 0.731$ ($SD = 0.20$) in the first half of block 1 and the highest performance rate of $M_{NoCue} = 0.851$ ($SD = 0.07$) in the second half of block 2.

**Table 1:** Memory task performance.

B	Condition effect	Experience effect	Interaction effect
1	$F(2,39) = 0.04$, $p = .96$, $\eta ^2_{p} < 0.01$	$F(1,39) = 0.08$, $p = .78$, $\eta ^2_{p} < 0.01$	$F(2,39) = 0.01$, $p = .99$, $\eta ^2_{p} < 0.01$
2	$F(2,39) = 0.04$, $p = .96$, $\eta ^2_{p} < 0.01$	$F(1,39) = 0.14$, $p = .71$, $\eta ^2_{p} < 0.01$	$F(2,39) = 1.36$, $p = .27$, $\eta ^2_{p} = 0.07$
3	$F(2,39) = 0.16$, $p = .85$, $\eta ^2_{p} < 0.01$	$F(1,39) = 2.52$, $p = .12$, $\eta ^2_{p} = 0.06$	$F(2,39) = 2.02$, $p = .15$, $\eta ^2_{p} = 0.09$

Tests statistics from the ANOVAs for block (B) 1, block 2 and block 3.

### Trust

**Figure 2:**
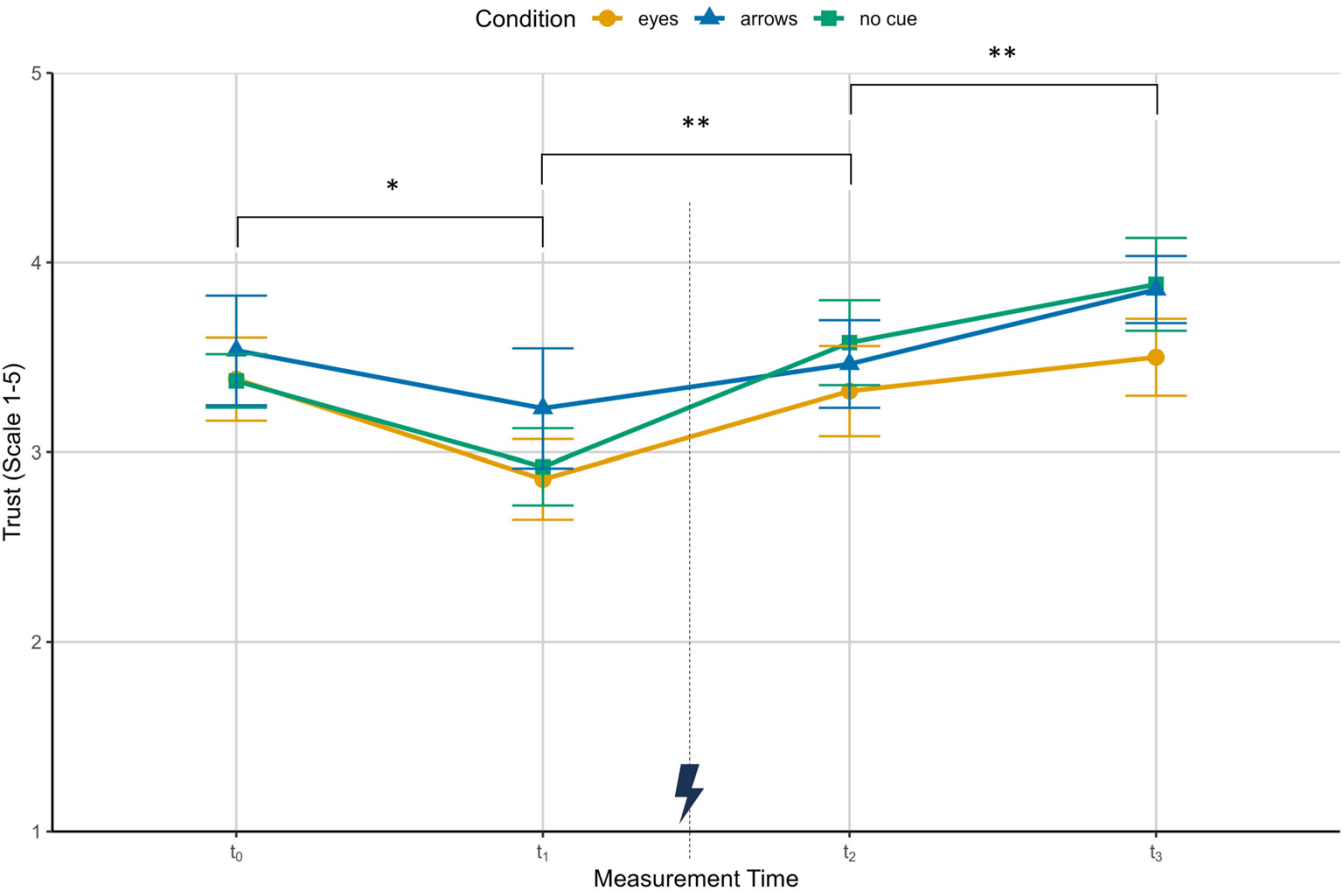
Subjective trust (scale: 1–5 with higher values indicating higher trust) for the three conditions across the initial trust measurement ($t_{0}$), trust after error-free interaction in block 1 ($t_{1}$), trust after failure experience in block 2 ($t_{2}$) and trust after error-free interaction in block 3 ($t_{3}$). Depicted are means and standard errors. The asterisks indicate significant time effects from $t_{0}$ to $t_{1}$ ($p = .018$), from $t_{1}$ to $t_{2}$ ($p = .008$) and from $t_{2}$ to $t_{3}$ ($p = .003$). The lightning bolt icon marks the occurrence of cue error trials.

As the trust questionnaire included a “no response” option that was treated as a missing value, some data were missing across all measurement points ($t_{0}$: 3 cases, $t_{1}$: 2 cases, $t_{2}$: 1 case, $t_{3}$: 1 case). For initial ($t_{0}$) and pre-failure trust ($t_{1}$), the analysis revealed a significant main effect of experience ($F(1,35) = 8.65$, $p = .018$, $\eta ^2_{p} = 0.20$) indicating a trust decrease following an error-free interaction block as can be seen in Fig. 2, while neither the condition effect ($F(2,35) = 0.28$, $p > .999$, $\eta ^2_{p} = 0.02$) nor the interaction reached significance ($F(2,35) = 0.20$, $p = .816$, $\eta ^2_{p} = 0.01$). Surprisingly, trust increased significantly from pre-failure experience in $t_{1}$ to post-failure experience in $t_{2}$ ($F(1,37) = 9.51$, $p = .008$, $\eta ^2_{p} = 0.20$), with no differences across conditions ($F(2,37) = 0.44$, $p >999$, $\eta ^2_{p} = 0.02$), nor an interaction ($F(2,37) = 0.54$, $p > .999$, $\eta ^2_{p} = 0.03$). This pattern persisted from $t_{2}$ to $t_{3}$ with a trust increase ($F(1,38) = 11.97$, $p = .003$, $\eta ^2_{p} = 0.24$) and again no condition ($F(2,38) = 0.64$, $p > .999$, $\eta ^2_{p} = 0.03$) or interaction effect ($F(2,38) = 0.55$, $p > .999$, $\eta ^2_{p} = 0.03$). In sum , we found an unexpected decline in trust after trials with no implemented cue errors and a trust increase following trials that included cue errors, with no systematic differences across conditions. This counterintuitive pattern suggests that factors beyond the presence of errors - such as participants’ evolving familiarity with the robot’s motion - may have shaped their trust evaluations (see Discussion).

### Visual attention allocation

#### Mean number of fixations in AOIs

Since the robotic arm and the display convey directional information, the analysis focused on these two areas of interest (AOI). The mean number of fixations on the display - showing either predictive cues or no cues - was calculated separately for the first six trials and the last six trials of a block. Overall, the lowest number of fixations per six trials was in the no cues condition $M_{NoCue} = 2.12$ ($SD = 1.15$). Though in general rather low, there were higher numbers of fixation in the conditions with predictive cues ($M_{Eyes} = 9.54$, $SD = 6.02$; $M_{Arrows} = 9.07$, $SD = 4.59$). This was also confirmed by the ANOVAs for block 1, 2 and 3, all revealing significant main effects of condition (Block 1: $F(2,39) = 9.29$, $p < .001$, $\eta ^2_{p} = 0.32$; Block 2: $F(2,39) = 7.63$, $p = .002$, $\eta ^2_{p} = 0.28$; Block 3: $F(2,39) = 10.22$, $p < .001$, $\eta ^2_{p} = 0.34$). In each block, Bonferroni corrected post hoc tests showed statistically significant differences between the no cues and eyes condition ($t_{Block 1}(39) = -3.64$, $p = .002$; $t_{Block 2}(39) = -3.75$, $p = .002$; $t_{Block 3}(39) = -3.88$, $p = .001$) as well as no cues and arrows condition ($t_{Block 1}(39) = -3.82$, $p = .001$; $t_{Block 2}(39) = -2.82$, $p = .002$; $t_{Block 3}(39) = -3.96$, $p < .001$). Eyes and arrows did not differ significantly ($t_{Block 1}(39) = -0.18$, $p > .999$; $t_{Block 2}(39) = 0.93$, $p > .999$; $t_{Block 3}(39) = -0.08$, $p > .999$) The number of fixations on the robotic arm was in general higher ($M_{NoCue} = 28.58$, $SD = 8.17$; $M_{Eyes} = 24.49$, $SD = 7.82$; $M_{Arrows} = 26.56$, $SD = 8.34$). However, in neither of the three blocks significant differences between the conditions emerged. The only significant effect was a decrease in the number of fixations within the first block. See Table 2 for all test statistics regarding the fixations on the robotic arm.

**Table 2:** ANOVA test statistics for the number of fixations on the robotic arm for block (B) 1, block 2 and block 3.

B	Condition effect	Experience effect	Interaction effect
1	$F(2,39) < 0.01$, $p > .99$, $\eta ^2_{p} < 0.01$	$F(1,39) = 7.75$, $p < .01$, $\eta ^2_{p} = 0.17$	$F(2,39) = 0.65$, $p = .53$, $\eta ^2_{p} = 0.03$
2	$F(2,39) = 1.31$, $p = .28$, $\eta ^2_{p} = 0.06$	$F(1,39) = 1.58$, $p = .22$, $\eta ^2_{p} = 0.04$	$F(2,39) = 3.00$, $p = .06$, $\eta ^2_{p} = 0.13$
3	$F(2,39) = 2.27$, $p = .12$, $\eta ^2_{p} = 0.10$	$F(1,39) = 0.30$, $p = .59$, $\eta ^2_{p} < 0.01$	$F(2,39) = 1.10$, $p = .34$, $\eta ^2_{p} = 0.05$

#### Mean fixation duration in AOIs

Similar to the mean number of fixations, the mean fixation duration was analyzed particularly for the AOI display and robotic arm. All ANOVA test statistics can be found in Table 3 for the display and Table 4 for the robotic arm. For the display, the mean duration of the fixation was around 229.09 ms ($SD = 70.49$ ms) for the no cue condition, around 251.49 ms ($SD = 73.54$ ms) for the arrows condition, and 258.93 ms ($SD = 554.60$ ms) for the eyes condition. No significant effects emerged.

**Table 3:** ANOVA test statistics for the fixation duration on the display for block (B) 1, block 2 and block 3.

B	Condition effect	Experience effect	Interaction effect
1	$F(2,35.38) = 1.00$, $p = .38$, $\eta ^2_{p} = 0.05$	$F(1,34.28) = 2.32$, $p = .14$, $\eta ^2_{p} = 0.06$	$F(2,34.20) = 0.46$, $p = .64$, $\eta ^2_{p} = 0.03$
2	$F(2,38.88) < 0.01$, $p > .99$, $\eta ^2_{p} < 0.01$	$F(1,37.34) = 0.06$, $p = .81$, $\eta ^2_{p} < 0.01$	$F(2,37.21) = 1.13$, $p = .34$, $\eta ^2_{p} = 0.06$
3	$F(2,38.18) = 1.28$, $p = .29$, $\eta ^2_{p} = 0.06$	$F(1,37.15) = 0.82$, $p = .37$, $\eta ^2_{p} = 0.02$	$F(2,36.32) = 1.10$, $p = .73$, $\eta ^2_{p} = 0.04$

The Satterthwaite approximation was used to estimate the denominator degrees of freedom.

For the robotic arm, the fixations were the longest in the condition without predictive cues ($M_{NoCue} = 356.96$ ms, $SD = 128.64$ ms), followed by the condition with arrows ($M_{Arrows} = 305.57$ ms, $SD = 80.05$ ms) and the condition with eyes on the display ($M_{Eyes} = 302.08$ ms, $SD = 88.48$ ms). However, those differences did not reach the statistical level of significance in neither of the three blocks (see Table 4). The only significant effect, was a significant increase in fixation duration within block 1 ($F(1,39) = 4.42$, $p = .04$, $\eta ^2_{p} = 0.10$).

**Table 4:** ANOVA test statistics for the fixation duration on the robotic arm for block (B) 1, block 2 and block 3.

B	Condition effect	Experience effect	Interaction effect
1	$F(2,39) = 1.10$, $p = .34$, $\eta ^2_{p} = 0.05$	$F(1,39) = 4.42$, $p = .04$, $\eta ^2_{p} = 0.10$	$F(2,39) = 2.16$, $p = .13$, $\eta ^2_{p} = 0.10$
2	$F(2,39) = 0.92$, $p = .41$, $\eta ^2_{p} = 0.05$	$F(1,39) = 1.80$, $p = .19$, $\eta ^2_{p} = 0.04$	$F(2,39) = 0.25$, $p = .78$, $\eta ^2_{p} = 0.01$
3	$F(2,39) = 1.54$, $p = .23$, $\eta ^2_{p} = 0.07$	$F(1,39) = 0.42$, $p = .52$, $\eta ^2_{p} = 0.01$	$F(2,39) = 0.35$, $p = .71$, $\eta ^2_{p} = 0.02$

#### Mean time to first target fixation

**Figure 3:**
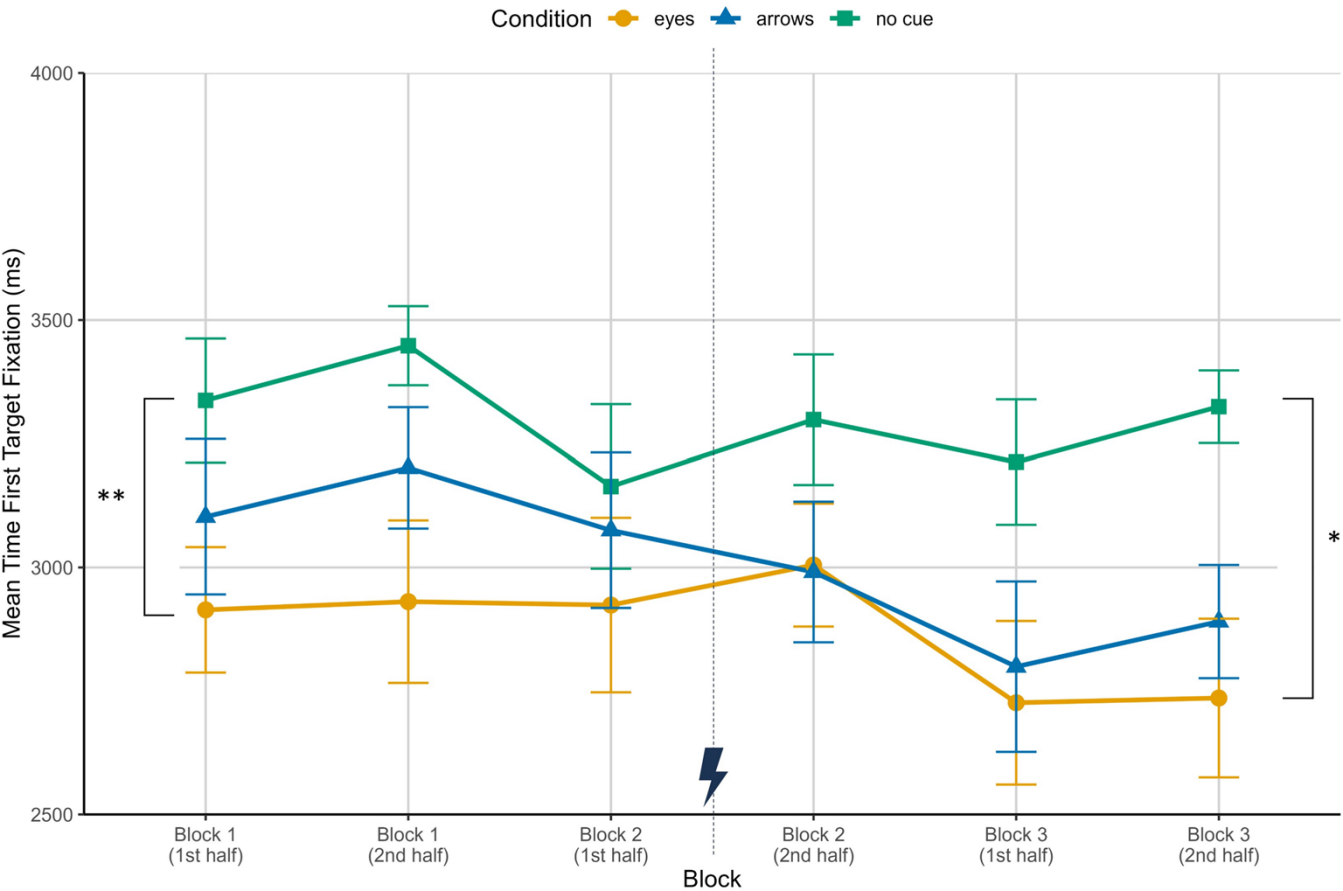
Mean time to first fixation of the correct target in milliseconds over the three experimental blocks, each split in the first six trials and the last six trials, for the three conditions eyes, arrows and no cue. Depicted are means and standard errors. The asterisks indicate a significant difference between the eyes and no cue condition in block 1 ($p = .006$) and in block 3 ($p = .013$). The lightning bolt icon marks the occurrence of cue error trials.

For the time to first target fixation, the mean duration from trial begin to the first fixation on the correct target was assessed. As can be seen in Fig. 3, participants in the condition without predictive cues consistently took the longest to identify the correct target ($M_{NoCue} = 3298$ ms, $SD = 350$ ms) followed by participants with arrows ($M_{Arrows} = 3010$ ms, $SD = 377$ ms) and the fastest identification of the correct target was achieved with eyes as predictive cues ($M_{Eyes} = 2873$ ms, $SD = 430$ ms). The ANOVA revealed a significant main effect of condition in block 1 ($F(2,39) = 5.57$, $p = .008$, $\eta ^2_{p} = 0.23$), with Bonferroni corrected post hoc tests showing that participants in the eyes condition were in fact significantly faster than in the no cues condition ($t(39) = 3.336$, $p = .006$), while the difference between no cues and arrows ($t(39) = 1.677$, $p = .305$) did not reach significance. The difference between eyes and arrows was also not significant ($t(39) = -1.597$, $p = .355$). Neither an effect of experience ($F(2,39) = 0.58$, $p = .447$, $\eta ^2_{p} = 0.02$) nor an interaction effect ($F(2,39) = 0.09$, $p = .912$, $\eta ^2_{p} < 0.01$) were found in block 1. In the second block, the ANOVA did not reveal any significant differences (Condition: $F(2,39) = 1.09$, $p = .35$, $\eta ^2_{p} = 0.05$; Experience: $F(1,39) = 0.28$, $p = .601$, $\eta ^2_{p} < 0.01$; Interaction: $F(2,39) = 0.63$, $p = .539$, $\eta ^2_{p} = 0.03$). For the third block, we found the same results as in block 1 - the predictive cue condition significantly influenced the time to first target fixation ($F(2,39) = 5.13$, $p = .011$, $\eta ^2_{p} = 0.21$). Again, target identification was significantly faster in the eyes condition compared to having no cues ($t(39) = 3.039$, $p = .013$), while participants in the arrows condition were only marginally significantly faster than in the no cues condition ($t(39) = 2.395$, $p = .065$). There was no significant difference between eyes and arrows ($t(39) = -0.644$, $p > .999$) and no significant experience ($F(2,39) = 0.96$, $p = .333$, $\eta ^2_{p} = 0.02$) or interaction effect ($F(2,39) = 0.19$, $p = .831$, $\eta ^2_{p} < 0.01$).

### Exploratory analyses

Workload was assessed as an exploratory measure. The analysis showed no significant differences across conditions. However, significant experience effects were found for several scales (mental and temporal demand, performance and frustration) showing reduced workload over time (e.g., mental demand: $F(2,78) = 4.56$, $p = .013$, $\eta ^2_{p} = 0.10$). In general, workload was perceived as quite high, e.g. temporal demand $M = 15.69$ ($SD = 2.50$) on a scale from 0-20. For the complete analysis of all workload scales, see the OSF.

Given that more than two-thirds of participants reported never or only occasionally using predictive cues, we explored objective cue usage by further analyzing the mean number of display fixations. A significant negative correlation was found between display fixations and mean prediction speed ($r = - .44$; $t(40) = -3.10$, $p = .004$), indicating that more frequent cue fixations were associated with faster predictions.

In addition, we found a slightly weaker but still moderate negative correlation ($r = - .38$; $t(40) = -2.56$, $p = .014$) between target prediction speed and memory task performance, suggesting that faster target predictions were associated with better performance in the memory task.

## Discussion

The primary objective of this research was to gain a better understanding of the influence of different predictive cues on performance, trust and visual attention allocation in a human–robot interaction with an industrial CoBot. For this purpose, we conducted a laboratory experiment where participants had to predict the movement target of a robotic arm. Depending on the condition, participants had additional directional information via predictive cues on the display of the robot. Participants either saw abstract anthropomorphic eyes, directional arrows or a black screen. After predicting the movement target, a memory task followed. We further investigated the effect of cue-errors by implementing a cue-related failure in one of the experimental blocks.

The manipulation check showed no significant differences in perceived anthropomorphism across conditions. In retrospect, this outcome is not entirely unexpected. The RoMo questionnaire assesses anthropomorphism across three dimensions: appearance, context, and movement. In our study, the robot’s overall design, context, and movement remained unchanged across conditions - the only variation was in the display of predictive cues. Since the eyes were deliberately designed to be highly abstract while still resembling basic human eye features (e.g., a pupil and sclera)^32^, their impact on perceived anthropomorphism was likely minimal. Therefore, compared to the conditions with arrows or no cues, the eye condition introduced only a subtle anthropomorphic element. As a result, any potential differences in perceived anthropomorphism may have been too small to be detected by the RoMo questionnaire. Still, prior research using the same predictive cues^26,32,33^ suggests that such cues can elicit gaze cueing and guide attention, supporting the effectiveness of the manipulation.

Contrary to our hypotheses, the predictive cues did not significantly influence the performance. Neither the eyes nor the arrows significantly improved action prediction compared to the no cues condition. This is not in line with prior research that found predictive cues to support more efficient action prediction^26,33^ and facilitate target identification^34^. One potential explanation could be that the predictive cues in our study followed a “gaze pattern” in order to appear more natural, while in previous research^26^, the cues statically fixated on the correct target. This gaze pattern could have reduced cue legibility and diminished potential benefits. Additionally, the errors in the cue conditions did not negatively impact action prediction. This might be explained by the fact that only a small number of participants (9/28) noticed the errors at all. Given the absence of significant differences in action prediction times between conditions, it is unsurprising that no significant differences emerged in memory task performance as the available time for the memory task was depending on the preceding prediction speed.

In terms of trust, the results did also differ from our expectations showing no significant differences. This might be explained by two factors: first, participants’ limited use of the cues, as indicated by their self-reports, and second, the lack of significant differences in perceived anthropomorphism between conditions. Nonetheless, previous studies have shown similar findings. For instance, Onnasch et al.^26^ found no trust-related effects of predictive cues, despite objective improvements in action prediction. Since predictability is often considered a key component of trust^38,51^, future research should investigate whether the absence of trust effects stems from specific features of this experimental setting or from broader limitations in how predictive cues influence trust. Furthermore, we found no evidence of a trust dissolution following the cue error. Given that so few participants recognized the error, this is not surprising - if an error goes unnoticed, it is unlikely to affect trust. Surprisingly, however, we observed a trust decline following the first interaction block, even though it was error-free. This may stem from participants misinterpreting the robot’s non-linear arm movements as erroneous. Although the robot’s movements were technically accurate, the trajectories were not always linear due to the robot’s degrees of freedom and the resulting joint configurations. Interestingly, some even attributed intentionality to this - one participant, for example, suggested the robot was “faking” a movement toward incorrect targets before selecting the correct one, while others said it “decided at the last moment” or moved “on purpose”. Over time, participants likely acclimated to the motion patterns, which may explain the observed increase in trust across all conditions in the later blocks. In sum, our hypotheses concerning trust and performance must be rejected.

However, looking at the eye tracking data, it could be shown that the eyes and arrows did attract attention as the mean number of display fixations was significantly higher in the predictive cue conditions compared to having no cues. Still, even though the difference is significant, the descriptive values of the display fixations were rather low for the predictive cue conditions as well. With about 9.54 fixations on the display in the eyes condition per six trials and about 9.04 fixations in the arrow condition, there were only on average between one and two fixations on the display per trial. This is in line with the self reported data of limited cue usage. Still, though predictive cue usage was rather low, we could see in the eye tracking data, what we had expected to see in the action prediction times - a significantly faster identification of the target when eyes were present compared to having no cues. On average, participants in the eyes condition were almost half a second faster in fixating the correct target than participants that had no predictive cues as additional direction information. This aligns with previous research suggesting that eye-like cues automatically trigger gaze cueing and attentional shifts^28,31^.

In our view, the most compelling explanation for the present set of findings (i.e. faster target identification but no corresponding improvement in action prediction) is twofold. First, participants appeared to rely on the cues less than expected. Second, even though the predictive cues had been validated in a series of previous studies^26,32,33^, where the robot eyes were shown to improve action prediction speed without reducing accuracy, we found that participants in the present study still experienced difficulties, particularly with interpreting spatial depth. The placement and coordinates of cues and targets were identical to those in the validated setup, but questionnaire feedback indicated that participants sometimes struggled to distinguish whether the cue referred to the front or back row of targets. Consequently, even when participants used the cues to identify the target, they often hesitated before making a selection, waiting for additional confirmation. This verification delay likely neutralized potential gains in overall action prediction time.

The fact that participants did not use the cues as much as anticipated is a surprising finding itself. We decided not to instruct the use of the predictive cues under the assumption that the cues would be used automatically as previous research suggested^26^. However, most participants did not rely on them. High workload, especially temporal workload, may have limited participants’ ability to notice or integrate additional directional information. In future work, explicitly instructing cue use may help to overcome this issue.

Two other results from this study merit comment. First, the exploratory analysis of the objective cue usage, measured by the fixation count on the display, suggested that higher cue usage was associated with faster action prediction. This indicates that the predictive cues provided useful directional information which, when attended to, could enhance prediction efficiency. Second, we found a moderate negative correlation between action prediction time and performance in the memory task, suggesting that faster action prediction was associated with better memory task performance. This indicates that the memory task may serve as a sensitive secondary measure of performance in our paradigm.

Although the present study indicates that predictive cues can aid action prediction in HRI when used, several limitations should be acknowledged.

First, limitations arise from the experimental setup. Self-reports showed that participants used the predictive cues less than we would have expected, which in turn likely contributed to the low rate of error detection - another limitation of the study. In addition, we implemented a “gaze-like” movement pattern for the predictive cues: they first pointed toward the target location, then briefly followed the robotic arm’s motion, and finally returned to the target. Although this pattern was intended to make the interaction feel more natural, it may have inadvertently reduced cue legibility. In reviewing the eye-tracking data, it became evident that some participants were still focused on the tablet used for the preceding memory task, when the cues made their initial shift toward the target, potentially missing the key predictive moment. Future studies should consider keeping the cues statically fixated on the target to ensure that the most important information - i.e., which area is being targeted next - is clearly conveyed. Moreover, participants reported difficulties in spatial depth estimation, which may have introduced uncertainty and contributed to the lack of significant effects in action prediction times.

Another limitation concerns the eye tracking data. Defining a dynamic AOI for the moving robotic arm was not feasible. Instead, we used a static AOI covering both the arm and the background it moved across. While we assume that gaze falling in this region is most likely directed at the robotic arm, given the absence of other visual stimuli, this approach introduces some degree of inaccuracy.

The study’s external validity also has some limitations. The use of a chin rest, necessary to ensure better eye-tracking data quality, created an artificial nature of the setting. However, while this compromises ecological validity, the inclusion of eye tracking was instrumental in revealing attentional dynamics that would have otherwise remained hidden. With that, we were able to identify a clear advantage of the predictive cues, particularly the eyes, in facilitating target identification. Additionally, it should be noted that participants were not explicitly instructed to consider the task as industrial, apart from the study being labeled as an industrial human–robot interaction. The industrial framing of the study primarily stems from the use of the CoBot Sawyer, a robotic arm widely deployed in production contexts. While the experimental setup could also be interpreted as a general joint attention task, our focus was on predictive cues (such as eyes and arrows) that are particularly relevant in industrial collaboration, where noise might limit the effectiveness of verbal communication, making non-verbal directional cues a promising means to facilitate coordination. The study does not attempt to reproduce an entire industrial workflow but instead intentionally isolates a core attentional mechanism with direct implications for collaborative industrial practice.

In conclusion, the study provides valuable insights into attentional mechanisms that might be crucial for practical industrial HRI applications - for example, that recognizing a robot’s intended target during pick-and-place tasks might be easier with anthropomorphic cues. However, there is still much work to be done before we fully understand how anthropomorphic predictive cues influence trust, task performance, and visual attention in HRI. Future studies should address current limitations by enhancing cue clarity, improving depth estimation, and instructing cue use. These findings contribute to the ongoing discourse on designing transparent and legible robotic behaviors - an increasingly important focus within the human-centric design paradigm of Industry 5.0.

## Methods

The study was pre-registered on the Open Science Framework (OSF Link) and approved by the ethics committee at the Department of Psychology and Ergonomics, Technische Universität Berlin. All research was performed in accordance with relevant guidelines/regulations and with the Declaration of Helsinki.

### Participants

A power analysis using G*Power^52^ (*f* = .25, power = .8, $\alpha$ = .05, groups = 3, measurements = 2, correlation = 0.5, nonsphericity correction = 1) determined a target sample size of 42. A total of 49 participants took part, but five were excluded due to low proficiency in German and two due to technical issues, leaving a final sample of *N* = 42, 14 per condition. The mean age was 27.67 (*SD* = 7.17); 61.9% were female, 33.3% male, 2.4% non-binary, and 2.4% preferred not to disclose their gender. Most participants (*n* = 39) were students in the fields of human factors or engineering. Over half of them (54.8 %) had prior experience with robots, e.g., through their study programme or other study participations. Participants were recruited via the university’s participant pool and received course credit. All provided informed consent.

### Design

We employed a 3 (predictive cue: abstract anthropomorphic eyes, directional arrows, no cues; between) $\times$ 3 (interaction experience: block 1 pre-failure, block 2 interaction with failure, block 3 post-failure; within) mixed design.

### Apparatus and task

**Figure 4:**
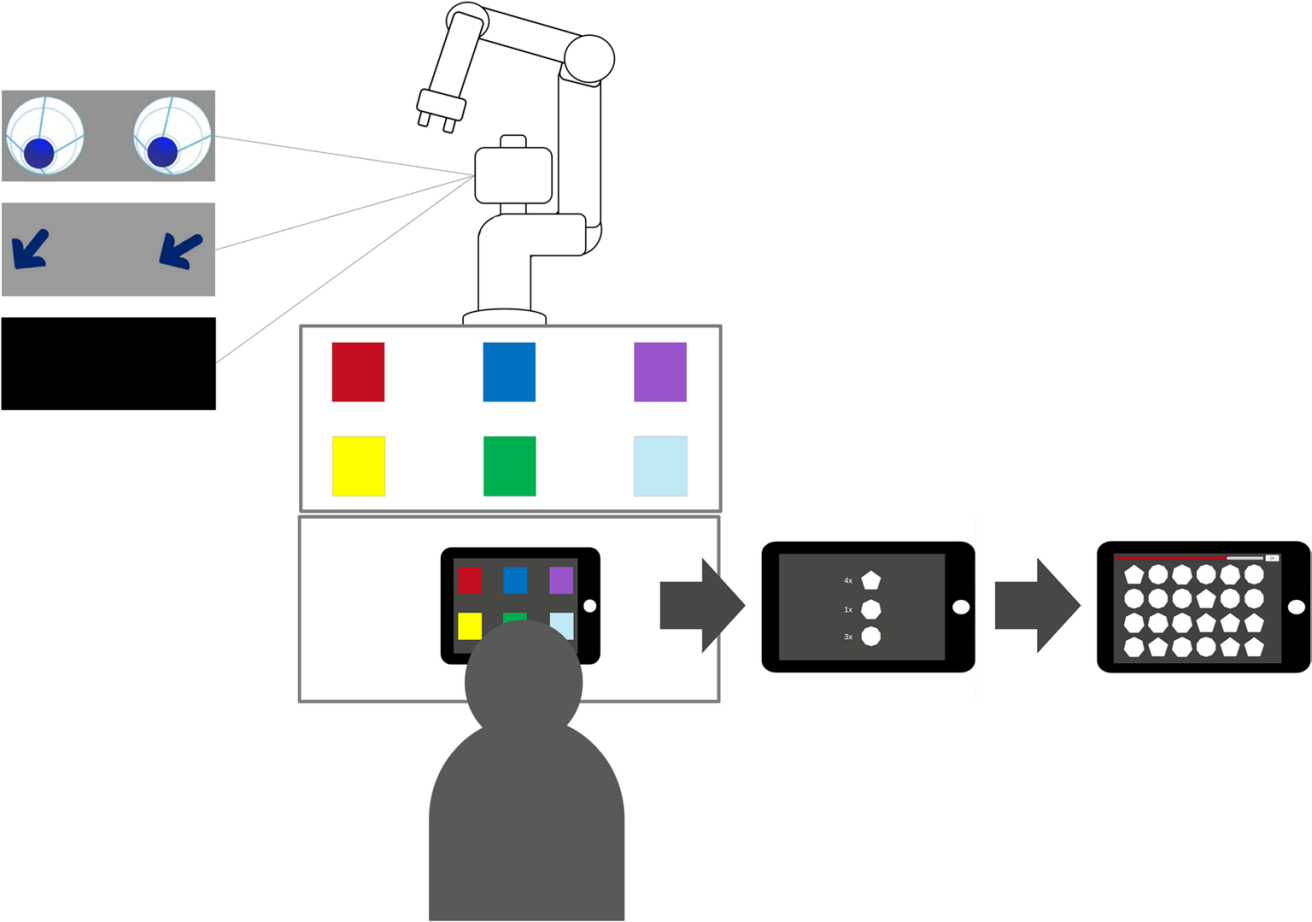
Experimental setting with the robot, showing on the display either eyes, arrows or no cues, the tablet showing the targets and the participant. After selecting the target, participants were shown the number of shapes that had to be selected from multiple shapes shown on the next screen. At the top of the screen a red bar shows the available time for the memory task.

The experimental paradigm was adapted from a larger research project on predictive cues in HRI referenced in^26^. Participants’ task was to predict the movement target of an industrial robotic arm. The robot used in this experiment was Sawyer from Rethink Robotics. Sawyer is an industrial CoBot that was designed for collaborative manufacturing tasks. It features a robotic arm with seven degrees of freedom and an integrated display. The robot is mounted on a pedestal, positioning the lower edge of the display at a height of 135 cm. Depending on the experimental condition, the display showed either abstract anthropomorphic eyes, directional arrows or no cues (i.e. a black screen). See Fig. 4 for the experimental conditions and the general setting.

Sawyer was positioned in front of a table, where six colored target squares (10cm x 10cm) were shown (red, blue, violet, yellow, green, cyan). Participants sat opposite, wearing the Neon eye tracking glasses from Pupil Labs. The Neon glasses are a mobile, binocular head-mounted eye tracker consisting of two infrared cameras that capture eye movements at 200Hz (resolution: 192 x 192 pixels) and a front-facing scene camera showing the field of view of the participant with 30Hz (resolution: 1600 x 1200 pixels, field of view: $132^{\circ } \times 81^{\circ }$)^53^. The eye tracking glasses are connected via USB-c cable to a mobile computing device (Motorola Edge 40 Pro) where the Neon companion app allows for operating the eye tracking (starting/stopping a recording, calibrating the glasses via gaze offset corrections). The recordings were uploaded and stored in the Pupil Cloud, a web-based eye tracking platform^53^. To ensure minimal head movements and improve tracking accuracy, participants used a chin rest. In front of them was a tablet that was connected via USB-c with the computer where the program for operating the robot was run. As tablet and robot had to work synchronously, a program on the computer simultaneously started the tablet when the robot was started. At the beginning of each trial, the six colored squares were shown on the tablet. Participants were instructed to predict the robotic arm’s target (one of the colored squares on the table) as quickly and accurately as possible by selecting the according square on their tablet. After the target selection, a memory task followed on the tablet as a second performance indicator. In this memory task, three geometric shapes (pentagon, heptagon, decagon) were shown with numbers summing to eight for two seconds, followed by a screen displaying multiple shapes where participants had to select the correct number of each of the shapes (see Fig. 4). The order and the number of the shapes were randomly created and then held constant for every participant. The available remaining time for the memory task depended on how quickly the correct target square was selected previously. The faster the target square was selected, the more time remained for the memory task within the trial.

Each trial started with the robotic arm in a neutral upwards position. In predictive cue conditions, eyes/arrows initially fixated on the robot’s gripper, then shifted to the target square one second before movement onset. During the movement of the robotic arm, the cues shifted and followed the motion of the arm for 600 ms before refocusing on the target again for the rest of the movement. The movement lasted six seconds, followed by a five-second return to the neutral position. All in all, each trial took in sum 11 seconds and ended in the same position as it began - the robotic arm in a neutral upwards position and the predictive cues pointing at the same location. See the OSF for a demonstration video.

Block 1 and block 3 included twelve error-free trials, with each square being targeted twice (total duration: 2 min 12 s). Block 2 introduced two additional error trials where predictive cues misdirected attention (e.g., cues pointed to cyan while the arm moved to yellow). This block comprised fourteen trials (six correct, two error, six correct; total duration: 2 min 34 s). Again, each square was targeted twice, apart from the yellow and cyan colored square which had one additional error-trial each. Trial order was randomized once and kept constant for all participants.

### Dependent measures

The main dependent variables were performance, trust, as well as visual attention allocation as an exploratory measure. All questionnaire-based measures were in German.

#### Manipulation check and control variables

Perceived anthropomorphism in appearance ($\omega _{\text {Appearance}} = .92$), movement ($\omega _{\text {Movement}} = .76$) and context ($\omega _{\text {Context}} = .88$) was assessed as a manipulation check using the Robot Morphology (RoMo-A) Questionnaire^54^. Each scale consists of three items (e.g., “How human-like is the robot’s external appearance?” for the appearance scale, “How human-like is the robot’s flow of movement?” for the movement scale, “How human-like is the robot’s task?” for the context scale) and is rated on a scale from 0% to 100%.

As control variables individual tendency to anthropomorphize robots (IDAQ-R) and mind perception were measured. The IDAQ-R is a customized questionnaire based on the short version of the Individual Differences in Anthropomorphism Questionnaire (IDAQ)^55^. The original IDAQ consists of 30 items that are focused on different agents that are commonly anthropomorphized (nonhuman animals, natural entities, spiritual agents and technological devices). A short version of the IDAQ already used in previous research^56^ consists only of the items related to technological devices. As we were interested specifically in the individual tendency to anthropomorphize robots, the questionnaire was adapted to refer to robots instead of technological devices like cars, computers or televisions. For instance, instead of “To what extent does a car have free will?”, “To what extent does a robot have free will?” was asked.The final questionnaire consists of four items rated on a scale from 0 (not at all) to 10 (very much) ($\omega = .81$).

To measure mind perception, a questionnaire based on Gray & Wegner (2012) was used ($\omega = .90$). The questionnaire includes four items, two corresponding to the dimension of experience (“This robot has the capacity to feel pain”, “This robot has the capacity to feel fear”) and two to agency (“This robot has the capacity to plan actions”, “This robot has the capacity to exercise self-control”). Items were rated on a 5-point Likert scale with 1 being “not at all” and 5 being “extremely”.

Furthermore, participants were asked whether they noticed any faulty behavior of the robot and, if so, to describe it. The responses were coded to determine whether the predictive cue errors during block 2 were detected. In addition, as a self-assessment of cue usage, participants rated how often they relied on the cues for the task on a scale from 1 (never) to 4 (always). Finally, demographic variables were recorded (gender, age, employment situation, previous experience with robots).

#### Performance

The main performance measure was prediction speed, i.e., the reaction time for selecting the correct target square in milliseconds. In addition, accuracy was assessed as the ratio of correct to total choices, resulting in values between 0 (no correct predictions) and 1 (all predictions correct). As a secondary performance measure, the performance in the memory task was registered. For this, a performance score was calculated based on the hit rate minus the false alarm rate resulting in scores between +1 (best possible score) and -1 (worst score).

#### Trust

Trust was measured using the “trust in automation” subscale of the “Trust in Automation” (TiA) questionnaire by Körber^57^, with two items (“I trust the system”, “I can rely on the system”). The items were rated on a 5-point Likert scale (1 = strongly disagree, 5 = strongly agree) with an additional “no response” option. Responses marked as “no response” were treated as missing values, as recommended by the author of the questionnaire^58^. Internal consistency ranged from $\alpha _{\text {t0}} = .71$ to $\alpha _{\text {t3}} = .84$.

#### Visual attention allocation

Eye tracking was used to explore visual attention allocation. Areas of Interest were defined for the areas of the robot providing directional information, i.e. the robotic arm and the display where predictive cues were shown as well as the target squares, and the tablet screen in front of the participants. Only fixations within these predefined AOIs were included in the analysis. Because of challenges associated with defining and analyzing dynamic AOIs, the AOI for the robotic arm was defined statically as the entire area in which the arm moved. Given the absence of other stimuli in this region, it was assumed that fixations in this area were directed toward the robotic arm. Fixations were automatically detected using the Pupil Labs fixation algorithm, with a lower threshold of 100 ms^59^. Three key measures were assessed: (1) the mean number of fixations per AOI, indicating its importance^59^; (2) mean fixation duration (ms), which can reflect either processing difficulty or engagement^59^. For those two measures, special focus was placed on the robotic arm and display AOIs to assess how predictive information was used; (3) mean time to first fixation of the correct target (ms), representing the speed of visual attention shifts to the targeted colored squares.

#### Exploratory workload measure

Additionally, workload was measured after each experimental block with the NASA-TLX^60^ as an exploratory measure to explore the influence of predictive cues on workload. It consists of six scales (mental, physical and temporal demand, performance, effort and frustration) rated on a 21-point scale from very low to very high.

### Procedure

**Figure 5:**
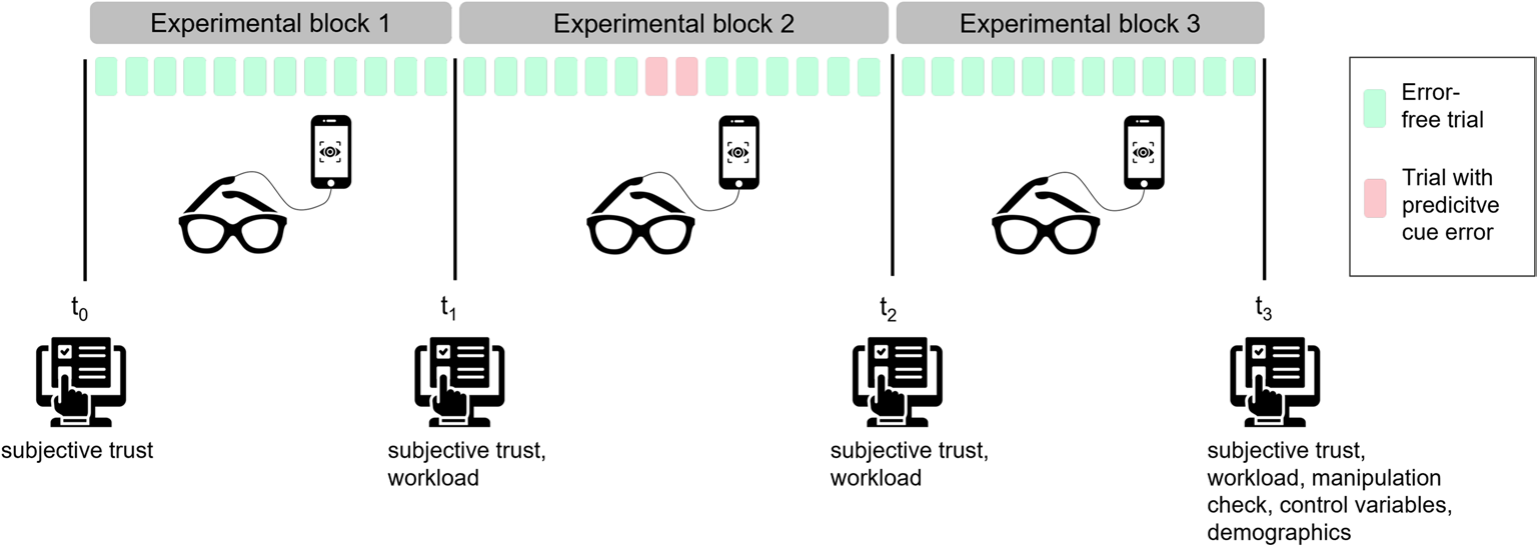
Experimental procedure consisting of three experimental blocks with 12 error free trials each (green squares) and two additional error trials in block two (red squares). During the blocks, eye tracking was recorded. Prior to the first block, and after each block subjective measures were assessed.

Upon arrival at the department’s laboratory, participants provided informed consent to take part in the study. They received their instructions via computer explaining the study procedure and their tasks. Then, initial trust was measured and eye tracking was calibrated. After this, participants practiced the memory task. They did not practice the task of predicting the robot’s target because the intuitive use of the predictive cues was of interest. If there were no further questions, the three experimental blocks started. Trust and workload were assessed after each block. After the final block, the manipulation check, control variables, and demographics were collected additionally to trust and workload. The study concluded with a debriefing. The entire session lasted about 40 minutes. See Fig. 5 for the procedure.

### Data analysis

Manipulation check and control variables were analyzed using one-way ANOVAs. To test performance hypotheses, we conducted one 3 (predictive cue) $\times$ 2 (experience: first 6 trials vs. last 6 trials) mixed ANOVA per experimental block. Blocks were split in half to examine potential within-block changes over time, particularly in response to the cue errors that occurred in block 2. This allowed us to specifically investigate how participants’ performance adapted from the first half of block 2 (pre-error) to the second half (post-error), providing insights into short-term adjustments to predictive cue reliability. The same subdivision approach was applied to all blocks to maintain analytical consistency.

For trust, separate 3 (predictive cue) $\times$ 2 (measurement time) mixed ANOVAs were conducted to assess trust formation (pre-interaction $t_0$ vs. post-block 1 $t_1$), trust dissolution (post-block 1 $t_1$ vs. post-failure $t_2$), and trust restoration (post-failure $t_2$ vs. post-block 3 $t_3$). Due to repeated analyses of trust at $t_1$ and $t_2$, Bonferroni correction was applied by multiplying the p-value by three for the three ANOVAs. Visual attention allocation was analyzed similarly to performance, using a 3 (predictive cue) $\times$ 2 (experience: first 6 trials vs. last 6 trials) mixed ANOVA per experimental block. Each workload dimension was examined with a 3 (predictive cue) $\times$ 3 (experience: post-block 1 $t_1$, post-block 2 $t_2$, post-block 3 $t_3$) mixed ANOVA. In general, in cases of significant main effects, Bonferroni-corrected post-hoc tests were performed.

## Data Availability

The dataset generated and analysed during the current study is available in the Open Science Framework: https://osf.io/tn8xd/. Also, the study was preregistered in the OSF: https://osf.io/fyg78.

## References

[1] Adel, A. Future of industry 5.0 in society: human-centric solutions, challenges and prospective research areas. *J. Cloud Comput.* **11**, 40 (2022). 10.1186/s13677-022-00314-5 PMC9454409 36101900

[2] Raffik, R., Sathya, R. R., Vaishali, V., Balavedhaa, S. et al. Industry 5.0: Enhancing human-robot collaboration through collaborative robots—a review. In *2023 2nd international conference on advancements in electrical, electronics, communication, computing and automation (ICAECA)*, 1–6 (IEEE, 2023).

[3] International Federation of Robotics. *World robotics report 2023* (Tech. Rep, IFR International Federation of Robotics, 2023).

[4] Hentout, A., Aouache, M., Maoudj, A. & Akli, I. Human-robot interaction in industrial collaborative robotics: a literature review of the decade 2008–2017. *Adv. Robot.* **33**, 764–799 (2019).

[5] Onnasch, L. & Hildebrandt, C. L. Impact of anthropomorphic robot design on trust and attention in industrial human-robot interaction. *ACM Trans. Human-Robot Interact. (THRI)* **11**, 1–24 (2021).

[6] Raviola, A. et al. Harmonic drive gear failures in industrial robots applications: an overview. *PHM Soc. Eur. Conf.* **6**, 11 (2021).

[7] Koppenborg, M., Nickel, P., Naber, B., Lungfiel, A. & Huelke, M. Effects of movement speed and predictability in human-robot collaboration. *Hum. Fact. Ergonom. Manuf. Serv. Ind.* **27**, 197–209 (2017).

[8] Sebanz, N. & Knoblich, G. Prediction in joint action: What, when, and where. *Top. Cogn. Sci.* **1**, 353–367 (2009). 25164938 10.1111/j.1756-8765.2009.01024.x

[9] Roesler, E., Manzey, D. & Onnasch, L. A meta-analysis on the effectiveness of anthropomorphism in human-robot interaction. *Sci. Robot.* **6**, eabj5425 (2021). 34516745 10.1126/scirobotics.abj5425

[10] Wiese, E., Metta, G. & Wykowska, A. Robots as intentional agents: using neuroscientific methods to make robots appear more social. *Front. Psychol.* **8**, 1663 (2017). 29046651 10.3389/fpsyg.2017.01663 PMC5632653

[11] Basili, P. et al. Inferring the goal of an approaching agent: A human-robot study. In *2012 IEEE RO-MAN: The 21st IEEE International Symposium on Robot and Human Interactive Communication*, 527–532 (IEEE, 2012).

[12] Onnasch, L. & Roesler, E. A taxonomy to structure and analyze human-robot interaction. *Int. J. Soc. Robot.* **13**, 833–849 (2021).

[13] De Graaf, M. M. & Allouch, S. B. Exploring influencing variables for the acceptance of social robots. *Robot. Auton. Syst.* **61**, 1476–1486 (2013).

[14] Darling, K. ’who’s johnny?’anthropomorphic framing in human-robot interaction, integration, and policy. *Anthropomorphic framing in human-robot interaction, integration, and policy (March 23, 2015). Robot Ethics* **2** (2015).

[15] Duffy, B. R. Anthropomorphism and the social robot. *Robot. Auton. Syst.* **42**, 177–190 (2003).

[16] Li, D., Rau, P. P. & Li, Y. A cross-cultural study: Effect of robot appearance and task. *Int. J. Soc. Robot.* **2**, 175–186 (2010).

[17] Riek, L. D., Rabinowitch, T.-C., Chakrabarti, B. & Robinson, P. How anthropomorphism affects empathy toward robots. In *Proceedings of the 4th ACM/IEEE international conference on Human robot interaction*, 245–246 (2009).

[18] Roesler, E., Onnasch, L. & Majer, J. I. The effect of anthropomorphism and failure comprehensibility on human-robot trust. *Proc. Hum. Factors Ergonom. Soc. Annu. Meet.* **64**, 107–111 (2020).

[19] Roesler, E., Vollmann, M., Manzey, D. & Onnasch, L. The dynamics of human-robot trust attitude and behavior-exploring the effects of anthropomorphism and type of failure. *Comput. Hum. Behav.* **150**, 108008 (2024).

[20] Onnasch, L. & Roesler, E. Anthropomorphizing robots: the effect of framing in human-robot collaboration. *Proc. Hum. Factors Ergonom. Soc. Annu. Meet.* **63**, 1311–1315. 10.1177/1071181319631209 (2019).

[21] Wainer, J., Feil-Seifer, D. J., Shell, D. A. & Mataric, M. J. Embodiment and human-robot interaction: A task-based perspective. In *RO-MAN 2007 - The 16th IEEE International Symposium on Robot and Human Interactive Communication*, 10.1109/roman.2007.4415207 (IEEE, 2007).

[22] Roesler, E., Manzey, D. & Onnasch, L. Embodiment matters in social HRI research: Effectiveness of anthropomorphism on subjective and objective outcomes. *ACM Trans. Hum. Robot Interact.*10.1145/3555812 (2022).

[23] Roesler, E., Naendrup-Poell, L., Manzey, D. & Onnasch, L. Why context matters: The influence of application domain on preferred degree of anthropomorphism and gender attribution in human-robot interaction. *Int. J. Soc. Robot.*10.1007/s12369-021-00860-z (2022).

[24] Goetz, J., Kiesler, S. & Powers, A. Matching robot appearance and behavior to tasks to improve human-robot cooperation. In *Proceedings of the 12th IEEE International Workshop on Robot and Human Interactive Communication (RO-MAN)*, 55–60 (IEEE, 2003).

[25] Yin, X. Influences of eye gaze cues on memory and its mechanisms: The function and evolution of social attention. *Front. Psychol.* **13**, 1036530 (2022). 36312084 10.3389/fpsyg.2022.1036530 PMC9614344

[26] Onnasch, L., Schweidler, P. & Wieser, M. Effects of predictive robot eyes on trust and task performance in an industrial cooperation task. In *Companion of the 2023 ACM/IEEE International Conference on Human-Robot Interaction*, 442–446 (2023).

[27] Emery, N. J., Lorincz, E. N., Perrett, D. I., Oram, M. W. & Baker, C. I. Gaze following and joint attention in rhesus monkeys (macaca mulatta). *J. Comp. Psychol.* **111**, 286 (1997). 9286096 10.1037/0735-7036.111.3.286

[28] Friebe, K., Samporová, S., Malinovská, K. & Hoffmann, M. Gaze cueing and the role of presence in human-robot interaction. In *International Conference on Social Robotics*, 402–414 (Springer, 2022).

[29] Admoni, H. & Scassellati, B. Robot gaze is different from human gaze: Evidence that robot gaze does not cue reflexive attention. In *Proceedings of the “Gaze in Human-Robot Interaction” Workshop at HRI* (2012).

[30] Tipples, J. Orienting to counterpredictive gaze and arrow cues. *Percept. Psychophys.* **70**, 77–87 (2008). 18306962 10.3758/pp.70.1.77

[31] Friesen, C. K., Ristic, J. & Kingstone, A. Attentional effects of counterpredictive gaze and arrow cues. *J. Exp. Psychol. Hum. Percept. Perform.* **30**, 319 (2004). 15053691 10.1037/0096-1523.30.2.319

[32] Onnasch, L., Kostadinova, E. & Schweidler, P. Humans can’t resist robot eyes-reflexive cueing with pseudo-social stimuli. *Front. Robot. AI* **9**, 848295 (2022). 37274454 10.3389/frobt.2022.848295 PMC10236938

[33] Onnasch, L., Schweidler, P. & Schmidt, H. The potential of robot eyes as predictive cues in hri-an eye-tracking study. *Front. Robot. AI* **10**, 1178433 (2023). 37575370 10.3389/frobt.2023.1178433 PMC10416260

[34] Boucher, J.-D. et al. I reach faster when i see you look: gaze effects in human-human and human-robot face-to-face cooperation. *Front. Neurorobot.* **6**, 3 (2012). 22563315 10.3389/fnbot.2012.00003 PMC3342577

[35] Staudte, M. & Crocker, M. W. Investigating joint attention mechanisms through spoken human-robot interaction. *Cognition* **120**, 268–291. 10.1016/j.cognition.2011.05.005 (2011). 21665198 10.1016/j.cognition.2011.05.005

[36] Lee, J. D. & See, K. A. Trust in automation: Designing for appropriate reliance. *Hum. Fact. J. Hum. Factors Ergonom. Soc.* **46**, 50–80. 10.1518/hfes.46.1.50_30392 (2004). 10.1518/hfes.46.1.50_30392 15151155

[37] Natarajan, M. & Gombolay, M. Effects of anthropomorphism and accountability on trust in human robot interaction. In *Proceedings of the 2020 ACM/IEEE international conference on human-robot interaction*, 33–42 (2020).

[38] Hancock, P. A. et al. A meta-analysis of factors affecting trust in human-robot interaction. *Hum. Fact. J. Hum. Factors Ergonom. Soc.* **53**, 517–527. 10.1177/0018720811417254 (2011). 10.1177/0018720811417254 22046724

[39] Hoff, K. & Bashir, M. A theoretical model for trust in automated systems. In *CHI ’13 Extended Abstracts on Human Factors in Computing Systems*, 115–120, 10.1145/2468356.2468378 (2013).

[40] Lewis, M., Sycara, K. & Walker, P. The role of trust in human-robot interaction. In Abbass, H. A., Scholz, J. & Reid, D. J. (eds.) *Foundations of Trusted Autonomy*, 135–159, 10.1007/978-3-319-64816-3_8 (Springer International Publishing, Cham, 2018).

[41] Desai, M., Kaniarasu, P., Medvedev, M., Steinfeld, A. & Yanco, H. Impact of robot failures and feedback on real-time trust. In *2013 8th ACM/IEEE International Conference on Human-Robot Interaction (HRI)*, 251–258 (IEEE, 2013).

[42] Flook, R. et al. On the impact of different types of errors on trust in human-robot interaction: Are laboratory-based hri experiments trustworthy?. *Interact. Stud.* **20**, 455–486 (2019).

[43] Hamacher, A., Bianchi-Berthouze, N., Pipe, A. G. & Eder, K. Believing in bert: Using expressive communication to enhance trust and counteract operational error in physical human-robot interaction. In *2016 25th IEEE international symposium on robot and human interactive communication (RO-MAN)*, 493–500 (IEEE, 2016).

[44] Zhao, Y., Zhu, Z. & Tang, B. Are highly anthropomorphic service robots more likely to be forgiven by customers after service failures? a mind perception perspective. *Int. J. Hosp. Manag.* **126**, 104103 (2025).

[45] Deubel, H. & Schneider, W. X. Saccade target selection and object recognition: Evidence for a common attentional mechanism. *Vision. Res.* **36**, 1827–1837 (1996). 8759451 10.1016/0042-6989(95)00294-4

[46] Just, M. A. & Carpenter, P. A. A theory of reading: from eye fixations to comprehension. *Psychol. Rev.* **87**, 329 (1980). 7413885

[47] Kompatsiari, K., Ciardo, F., De Tommaso, D. & Wykowska, A. Measuring engagement elicited by eye contact in human-robot interaction. In *2019 IEEE/RSJ International Conference on Intelligent Robots and Systems (IROS)*, 6979–6985 (IEEE, 2019).

[48] Lu, Y. & Sarter, N. Modeling and inferring human trust in automation based on real-time eye tracking data. *Proc. Hum. Fact. Ergonom. Soc. Annu. Meet.* **64**, 344–348 (2020).

[49] Patton, C. E. & Wickens, C. D. The relationship of trust and dependence. *Ergonomics* **67**, 1535–1552 (2024). 38725397 10.1080/00140139.2024.2342436

[50] Zhang, Y., Yadav, A., Hopko, S. K. & Mehta, R. K. In gaze we trust: Comparing eye tracking, self-report, and physiological indicators of dynamic trust during hri. In *Companion of the 2024 ACM/IEEE International Conference on Human-Robot Interaction*, 1188–1193 (2024).

[51] Law, T. & Scheutz, M. Trust: Recent concepts and evaluations in human-robot interaction. *Trust Human Robot Interact.* 27–57 (2021).

[52] Faul, F., Erdfelder, E., Lang, A.-G. & Buchner, A. G* power 3: A flexible statistical power analysis program for the social, behavioral, and biomedical sciences. *Behav. Res. Methods* **39**, 175–191 (2007). 17695343 10.3758/bf03193146

[53] Baumann, C. & Dierkes, K. *Neon accuracy test report*10.5281/zenodo.10420388 (2023).

[54] Roesler, E., Zur Kammer, K. & Onnasch, L. Multidimensionale fragebögen zur erfassung der wahrgenommenen robotermorphologie (romo) in der mensch-roboter-interaktion. *Zeitschrift für Arbeitswissenschaft* **77**, 609–628 (2023).

[55] Waytz, A., Cacioppo, J. & Epley, N. Who sees human? the stability and importance of individual differences in anthropomorphism. *Perspect. Psychol. Sci. J. Assoc. Psychol. Sci.* **5**, 219–232. 10.1177/1745691610369336 (2010). 10.1177/1745691610369336 PMC4021380 24839457

[56] Roesler, E., Heuring, M. & Onnasch, L. (hu) man-like robots: The impact of anthropomorphism and language on perceived robot gender. *Int. J. Soc. Robot.* **15**, 1829–1840 (2023). 10.1007/s12369-023-00975-5 PMC10027596 37359431

[57] Körber, M. Theoretical considerations and development of a questionnaire to measure trust in automation. In *Proceedings of the 20th Congress of the International Ergonomics Association (IEA 2018) Volume VI: Transport Ergonomics and Human Factors (TEHF), Aerospace Human Factors and Ergonomics 20*, 13–30 (Springer, 2019).

[58] Körber, M. Manual for the questionnaire “trust in automation” (tia) (2020). Accessed: 2025-05-02.

[59] Poole, A. & Ball, L. J. Eye tracking in hci and usability research. In Ghaoui, C. (ed.) *Encyclopedia of human computer interaction*, 211–219, 10.4018/978-1-59140-562-7.ch034 (IGI Global Scientific Publishing, 2006).

[60] Hart, S. G. & Staveland, L. E. Development of nasa-tlx (task load index): Results of empirical and theoretical research. In *Advances in psychology*, vol. 52, 139–183 (Elsevier, 1988).

